# Uncovering the repertoire of fungal secondary metabolites: From Fleming's laboratory to the International Space Station

**DOI:** 10.1080/21655979.2017.1341022

**Published:** 2017-07-12

**Authors:** Tomasz Boruta

**Affiliations:** Lodz University of Technology, Faculty of Process and Environmental Engineering, Department of Bioprocess Engineering, Lodz, Poland

**Keywords:** biosynthetic gene clusters, fungi, microgravity, radiation, secondary metabolism

## Abstract

Fungi produce a variety of secondary metabolites (SMs), low-molecular weight compounds associated with many potentially useful biologic activities. The examples of biotechnologically relevant fungal metabolites include penicillin, a β-lactam antibiotic, and lovastatin, a cholesterol-lowering drug. The discovery of pharmaceutical lead compounds within the microbial metabolic pools relies on the selection and biochemical characterization of promising strains. Not all SMs are produced under standard cultivation conditions, hence the uncovering of chemical potential of investigated strains often requires the use of induction strategies to awake the associated biosynthetic genes. Triggering the secondary metabolic pathways can be achieved through the variation of cultivation conditions and growth media composition. The alternative strategy is to use genetic engineering to activate the respective genomic segments, e.g. by the manipulation of regulators or chromatin-modifying enzymes. Recently, whole-genome sequencing of several fungi isolated from the Chernobyl accident area was reported by Singh et al. (Genome Announc 2017; 5:e01602–16). These strains were selected for exposure to microgravity at the International Space Station. Biochemical characterization of fungi cultivated under extreme conditions is likely to provide valuable insights into the adaptation mechanism associated with metabolism and, possibly, a catalog of novel molecules of potential pharmaceutical importance.

## Introduction

Recently, whole-genome sequencing of several fungal strains isolated from the area of Chernobyl power plant accident was reported.^1^ These fungi were selected to be exposed to microgravity at the International Space Station (ISS). Their physiologic characterization is highly anticipated, particularly in the context of metabolism, as the isolated radio-tolerant strains cultivated under the conditions of altered gravity could be the source of previously unknown valuable chemical compounds. The idea of conducting the growth experiments in space represents a promising approach of taking advantage of yet unexplored, often extreme environments to reveal the biosynthetic capabilities of microbial cells.^2^

### Fungal secondary metabolism

Fungi produce a wide variety of molecules referred to as secondary metabolites (SMs), e.g., polyketides, non-ribosomal peptides and terpenes.^3^ While not directly involved in fundamental metabolic processes of growth and energy generation, SMs display an array of biologic activities that contribute to the survival of the producing organism in an occupied ecological niche. Due to the exhibited bioactivity, many SMs can be regarded as promising leads for drug development efforts.^4^ Penicillin (a β-lactam antibiotic) and lovastatin (a cholesterol-lowering drug) are some examples of pharmaceutical significance and industrial impact associated with the application of SMs. Commercially important SMs of fungal origin are listed in Table 1 (for reviews, see refs.^5,6^).

**Table 1.** Examples of biotechnologically relevant fungal secondary metabolites.

Secondary metabolite	Producing fungus	Application
astaxanthin	*Phaffia rhodozyma*	pigment
*β*-carotene	*Blakeslea trispora*	pigment
cephalosporin C	*Acremonium chrysogenum*	resource for the production of cephalosporins
cyclosporine A	*Tolypocladium inflatum*	immunosuppressant
gibberelic acid	*Gibberella fujikuroi*	plant growth regulator
griseofulvin	*Penicillium griseofulvum*	antifungal agent
lovastatin	*Aspergillus terreus*	cholesterol-lowering drug
monascin, ankaflavin, monascorubrin, rubropunctatin	*Monascus* sp.	pigments
mycophenolic acid	*Penicillium* sp	immunosuppressant
penicillin G	*Penicillium rubens*	antibiotic
Taxol	*Taxomyces andreanae*	anticancer drug

The discovery of penicillin by Alexander Fleming, who observed the ongoing lysis of staphylococcus colonies on a plate contaminated by a filamentous fungus, provided the basis for what was to become a major scientific and medical breakthrough in treating bacterial infections.^7^ Several factors played a role in this serendipitous discovery. Firstly, the repertoire of SMs is a unique feature of every fungal strain and, importantly, only a limited number of fungi produce penicillin. The fungus that found its way to the Fleming's plate was *Penicillium rubens*, a species equipped with a penicillin biosynthetic gene cluster (BGC), a genomic segment encoding a set of proteins collectively responsible for the biosynthesis of this antibiotic.^8,9^ Secondly, it should be emphasized that the formation of a particular SM proceeds under specific conditions. In other words, it is typical for a fungus to reveal only a fraction of its chemical diversity under a given set of environmental cues.^10^ Fortunately, the nutrients and stimulatory signals to which *P. rubens* was exposed in the Fleming's laboratory triggered the biosynthetic machinery leading to penicillin formation and secretion. Hence, both the biochemical capabilities of the producer itself and the encountered environmental stimuli determine whether the given SM is produced or not. Notably, many BGCs remain silent under standard laboratory cultivation conditions and, as a consequence, the corresponding metabolites are not formed. In such cases, one may resort to special methods developed for the activation of secondary metabolic pathways.^11^ In the search for valuable compounds the challenge is not only to select the promising strains but also to effectively induce the biosynthesis of as many SMs as possible. This results in the expansion of observed metabolic pool and increases the chance of finding the molecule displaying the desired bioactivity. The study leading to the discovery of statins, the cholesterol-lowering SMs, represents a classic example of a successful pharmaceutical-oriented project involving fungal metabolism. Statins were isolated in the course of extensive screening experiments encompassing thousands of culture broths. These efforts fueled the development of natural, semi-synthetic and synthetic statin drugs prescribed to lower cholesterol levels.^12,13^

### Exploring the metabolic repertoire of fungi: cultivation-based and genetic engineering-based methods

One of the key aspects of finding novel bioactive SMs is to uncover the true biosynthetic potential of the examined organism. A number of effective approaches for the activation of SMs biosynthesis can be found in literature (for review, see refs.^14-20^). They involve the cultivation of target microorganisms under a variety of growth conditions or introducing genetic modifications to induce silent clusters. Whereas genetic manipulations are successfully applied for rational and targeted pathway activation, cultivation-based strategies aim at recreating the environmental signals that trigger cellular response leading to SMs production. Manipulation of global and cluster-specific regulators and altering the chromatin structure by deletion of histone deacetylases are the examples of genetic interventions conducted to trigger secondary metabolic routes. Alternatively, the BGC of interest can be heterologously expressed in various hosts, e.g., in yeast. While very effective, these approaches require the availability of sequence data, molecular engineering tools and methodology adapted to modify the genome of a particular species. In contrast, the widely-applied approach relying on the manipulations of growth media composition, physical parameters or cultivation strategies is relatively simple to follow, albeit typically more time-consuming. The encountered environmental signals influence fungal cells on multiple levels, including regulatory, signaling and metabolic pathways, developmental processes, morphology, adaptation and stress response.^10^ One of the common approaches to induce secondary metabolism is to subject the cells to stress, e.g., oxidative or osmotic, which can elicit a myriad of molecular defensive mechanisms accompanied by SMs production.

The cultivation-based and genetic engineering-based approaches can be seen as complementary. Whenever the combination of signals required to awake a particular set of BGCs is unlikely to be encountered in the course of laboratory growth experiments, even an extensive screening procedure involving an array of conditions may prove insufficient to reveal the metabolic potential of a fungus under study. In such cases, genomic manipulations are invaluable for exploring fungal chemical diversity. However, it may be very difficult to execute the bioengineering concepts when working with the strains less amenable to genetic manipulations. Furthermore, the cultivation-based methods can be readily applied for newly isolated, uncharacterized organisms, for which genome sequencing has not been yet performed.

### Secondary metabolism under extreme conditions

Cultivation of radiation-resistant fungi under microgravity at the International Space Station represents one of the recent efforts toward identifying new molecules of biotechnological relevance.^1^ As depicted in Table 2, isolating fungal strains from extreme environments was shown to be an effective strategy to discover novel SMs (for a review, see ref.^2^).

**Table 2.** Examples of fungi and novel secondary metabolites isolated from extreme environments (for a review, see ref.^2^).

Isolated fungus	Location	Isolated secondary metabolites
*Alternaria raphani*	Hongdao sea salt field, People's Republic of China	alternarosides A-C; alternarosin A^23^
*Aspergillus clavatus* C2WU	sulfur-rich hydrothermal vents, Taiwan	clavatustides A and B^24^
*Aspergillus westerdijkiae* DFFSCS013	deep-sea sediment (2918 m depth), South China Sea	circumdatins K and L; 5-chlorosclerotiamide; 10-*epi*-sclerotiamide; aspergilliamide B^25^
*Chaetomium globosum*	Sonoran Desert, Arizona, USA	globosumones A-C^26^
*Malbranchea sulfurea*	Sihchong River Hot Springs Zone, Taiwan	malbranpyrroles A–F^27^
*Penicillium* sp	Berkeley Pit (acid mine waste lake of high metal content), USA	berkeleydione; berkeleytrione; berkelic acid^28,29^
*Penicillium citrinum* HGY1–5	crater ash from the extinct volcano Huguangyan, People's Republic of China	C25 steroid isomers with bicyclo[4.4.1]A/B rings^30^
*Penicillium crustosum* PRB-2	deep-sea sediment (526 m depth), Antarctica	penilactones A and B^31^
*Pleurostomophora* sp	Berkeley Pit (acid mine waste lake of high metal content), USA	berkchaetoazaphilones A-C; berkchaetorubramine^32^
*Talaromyces thermophilus* (strains YM1–3 and YM3–4)	Tengchong hot springs, People's Republic of China	talathermophilins A-D^33,34^

The microbial growth in the area of Chernobyl power plant accident is inevitably associated with adaptation to extreme conditions and facing immense selective pressure.^21,22^ To survive and thrive in this highly challenging environment fungal strains need to exhibit unique metabolic characteristics associated with adaptation. Subjecting them to microgravity requires further reshaping of their biochemical machinery, possibly with the participation of previously unknown metabolites. When applied in concert, the 2 sources of stress, namely increased radiation and altered gravity, may induce cellular responses which were previously not observed in SM-oriented studies. In addition to the search for novel bioactive molecules, the elucidation of underlying adaptation mechanisms represents a great scientific challenge. Analyzing how fungal metabolism responds to space conditions will surely bring a plethora of biologic insights into the field of fungal SM and biology in general. While the regulation of SM is still far from being deciphered, the growth experiments conducted in space could fuel the advancement in the field by contributing valuable observations and, importantly, rich experimental data sets. Furthermore, it is likely that the scope of metabolic research in space will expand to incorporate a large number of bacterial and fungal species. It remains to be seen whether the microorganisms orbiting the Earth can provide us with the successors of penicillin, lovastatin and other relevant molecules of microbial origin.
